# Multiple functions of HMGB1 in cancer

**DOI:** 10.3389/fonc.2024.1384109

**Published:** 2024-04-25

**Authors:** Guangyao Lv, Menglin Yang, Keke Gai, Qiong Jia, Zhenzhen Wang, Bin Wang, Xueying Li

**Affiliations:** ^1^ Department of Pharmacy, Binzhou Medical University Hospital, Binzhou, Shandong, China; ^2^ Quality Management Department, Marine Biomedical Research Institute of Qingdao, Qingdao, China; ^3^ School of Health, Binzhou Polytechnic, Binzhou, China

**Keywords:** HMGB1, cancer, chemotherapy resistance, autophagy, immunotherapy

## Abstract

High mobility group box 1 (HMGB1) is a nuclear DNA-binding protein with a dual role in cancer, acting as an oncogene and a tumor suppressor. This protein regulates nucleosomal structure, DNA damage repair, and genomic stability within the cell, while also playing a role in immune cell functions. This review comprehensively evaluates the biological and clinical significance of HMGB1 in cancer, including its involvement in cell death and survival, its potential as a therapeutic target and cancer biomarker, and as a prosurvival signal for the remaining cells after exposure to cytotoxic anticancer treatments. We highlight the need for a better understanding of the cellular markers and mechanisms involved in the involvement of HMGB1in cancer, and aim to provide a deeper understanding of its role in cancer progression.

## 1 Introduction

High mobility group box 1 (HMGB1) is a highly conserved non-histone chromatin-associated nuclear DNA-binding protein that plays an essential role in regulation of various cellular processes, including nucleosomal structure, DNA damage repair, and genomic stability (1, 2). Since its discovery and isolation from calf thymus in 1973, HMGB1 has been the subject of extensive research for over half a century (3). This protein also participates in various immunological functions, acting as a danger-associated molecular pattern (DAMP) triggering various immune responses (4). HMGB1 has also been implicated in different diseases, including cancer (5).

The involvement of HMGB1 in cancer is complex, and its precise mechanisms are not yet fully understood. Studies have shown that HMGB1 plays a dual role in cancer by acting as an oncogene and tumor suppressor (6). HMGB1 regulates cellular death and survival pathways, as well as contributes to various stages of tumor progression, including proliferation, invasion, and metastasis. HMGB1 overexpression has been associated with a poor prognosis in various types of cancer (e.g., breast, lung, and colorectal). A study has discovered a highly trustworthy HMGB1 protein structure model in mice, which could facilitate the docking and prediction of anticancer drugs, such as CGA conformers, that bind to the active target site of HMGB1, potentially leading to the development of a universal anticancer drug effective against various cancer types (7). Therefore, it is important to understand the role of HMGB1 in the cancer diagnosis, prognosis, and treatment.

This review aimed to provide a comprehensive overview of the role of HMGB1 in cancer. We describe the mechanisms underlying the contribution of HMGB1 to tumor development, progression, and metastasis, together with its potential role as a biomarker and therapeutic target in cancer. This review also highlights the current state of knowledge and gaps in our understanding of the role of HMGB1 in cancer, providing a foundation for future research and the development of novel therapeutic strategies.

## 2 Structural variability of HMGB1 protein

**Figure 1:**
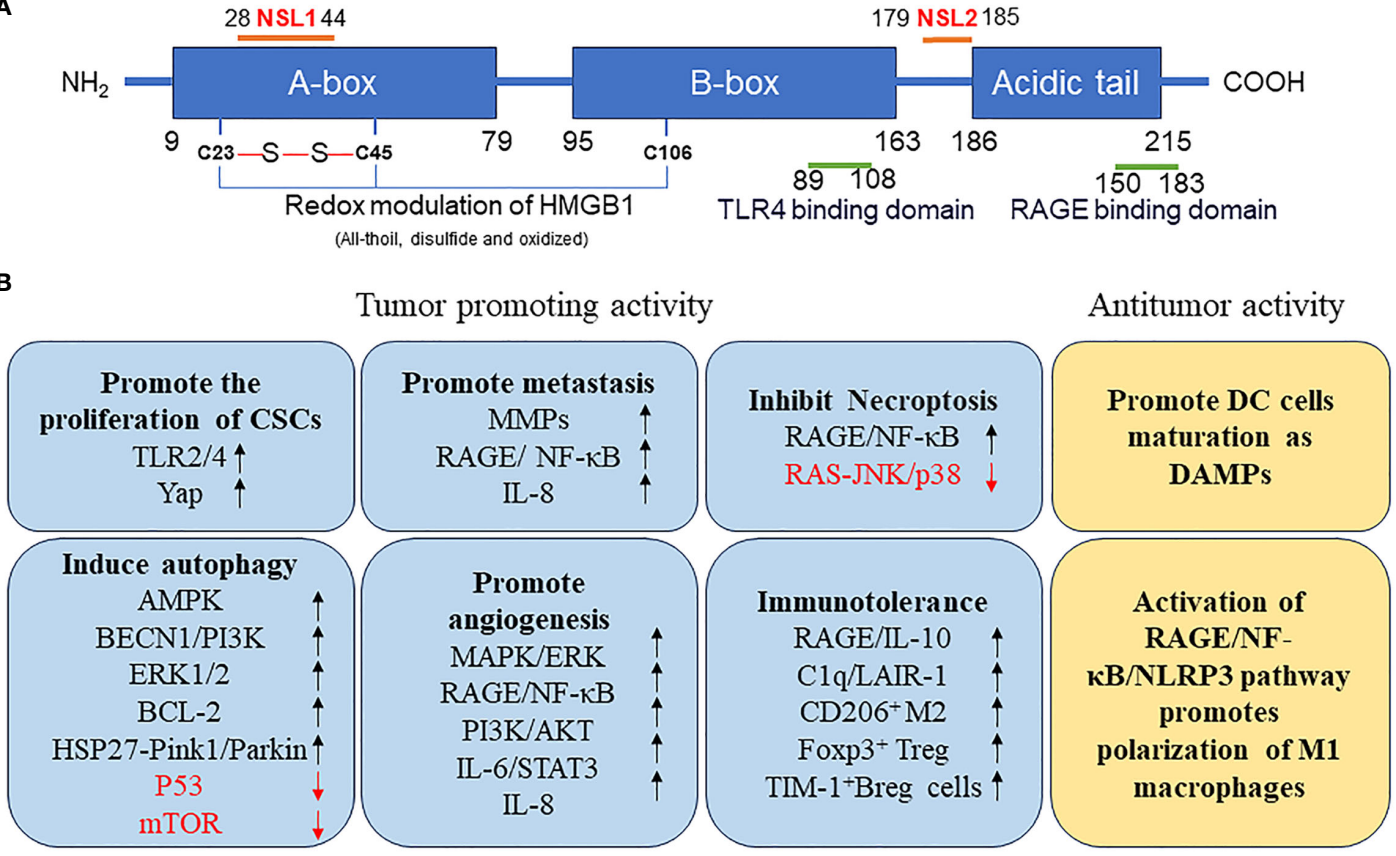
**(A)** Structure of HMGB1 protein. **(B)** Function of HMGB1 protein in tumor. AMPK, AMP-activated protein kinase; BCL2, B-cell CLL/lymphoma 2; BECN, beclin; Breg, regulatory B; CSCs, cancer stem cells; DAMP, danger-associated molecular pattern; DC, dendritic cell; ERK, extracellular signal-regulated kinase; FOXP3, forkhead box P3; HMGB1, high mobility group box 1; HSP27, heat-shock protein 27; IL, interleukin; JNK, c-Jun N-terminal kinase; LAIR1, leukocyte-associated immunoglobulin like receptor 1; MMPs, matrix metallopeptidases; mTOR, mechanistic target of rapamycin kinase; NF-κB, nuclear factor-kappa B; NLRP3, NLR family pyrin domain containing 3; NLS, nuclear localization signals; TP53, tumor protein 53; PI3K, phosphatidylinositol 3 kinase; PINK1, PTEN induced kinase 1; RAGE, receptor for advanced glycation end products; TIM1, T-cell immunoglobulin mucin family member 1; TLR4, toll-like receptor 4; YAP, Yes1 associated transcriptional regulator.

HMGB1 is a highly conserved single-chain polypeptide consisting of 215 amino acid residues. Its N-terminal region is enriched with positively charged lysine residues, whereas its C-terminal region is predominantly composed of negatively charged aspartic acid and glutamic acid, collectively referred to as the acidic tail. The protein structure of HMGB1 spans from the amino terminus to the carboxy terminus, encompassing an A box (residues 9–79), a B box (residues 95–163), and a receptor-binding motif (residues 186–215), which are exclusively composed of glutamic acid and aspartic acid. Crucially, HMGB1 contains two essential nuclear localization signals (NLS), specifically located at amino acids 28–44 (NLS1) and 179–185 (NLS2). These NLSs are responsible for the nuclear localization of HMGB1; moreover, they regulate its translocation between the nucleus and the cytoplasm upon post-translational modifications, such as phosphorylation and acetylation. Additionally, HMGB1 features a toll-like receptor 4 (TLR4)-binding site (residues 89–108) and a receptor for advanced glycation end products (RAGE)-binding site (residues 150–183), which contribute to its diverse functional interactions (**Figure 1A**). The structural hallmark of HMGB1 lies in its three distinct domains: two HMGBs, an NLS, and an acidic tail. These domains, together with the various binding sites, allow HMGB1 to perform vital roles in cellular processes, including immune regulation and tumor development (**Figure 1B**).

HMGB1 exhibits structural variations depending on its redox state and the presence or absence of disulfide bonds. These structural changes directly affect biological functions (1).

In their reduced state, specific cysteine residues of HMGB1, such as those located at positions 23, 45, and 106, remain in the reduced form. Reduced HMGB1 exhibits chemotactic activity, attracting leukocytes without relying on traditional cytokines or chemokines. Furthermore, the reduced state of HMGB1 opposes oxidation due to reactive oxygen species (ROS), maintaining its structural stability and allowing normal execution of its biological functions. This state also contributes to the promotion of repair of damaged tissue and regeneration and enhances the phagocytic activity of phagocytic cells and the autophagic activity in neighboring cancer cells.

In contrast, when HMGB1 is in its oxidized state, cysteine residues are oxidized, leading to the loss of chemotactic and cytokine-inducing activities. Oxidation is particularly common in highly acidic environments. The oxidized form of HMGB1 exhibits suppressed immunogenic function, and its intramolecular interactions can also undergo changes, that affect the conformational structure and stability of HMGB1. Such structural alterations can potentially affect the role of HMGB1 in cell signaling and immune responses.

Specifically, the presence of disulfide bonds in HMGB1 alters its structure. When the disulfide bond is located within the Box-A region (between cysteines at positions 23 and 45), the cytokine-induced activity of HMGB1 is suppressed. This structural change may affect the interactions between HMGB1 and other molecules, thereby influencing its function in cell signaling and immune responses.

## 3 Role of HMGB1 in inflammation

Elevated HMGB1 levels have been observed in patients with various inflammatory conditions, including mechanical trauma, stroke, acute myocardial infarction, acute respiratory distress, and liver transplantation. This protein is actively released from cells during injury or stress, and its extracellular presence can trigger inflammatory cascades (8).

In hepatic ischemia–reperfusion injury, HMGB1 levels increase early after reperfusion and persist for a prolonged periods (9). This increase in HMGB1 levels contributes to liver damage by promoting the release of pro-inflammatory cytokines. Administration of soluble RAGE or neutralizing antiHMGB1 antibody attenuates liver damage, further supporting the role of HMGB1 in this inflammatory process. TLR4, a HMGB1 receptor, is also involved in hepatic ischemia–reperfusion injury, and TLR4-deficient mice are protected from this injury. TLR4 mediates HMGB1-induced inflammation and regulates the HMGB1 secretion. Hypoxia, a common feature of ischemia–reperfusion, induces the active release of HMGB1 from hepatocytes through a TLR4-dependent mechanism involving ROS production and calcium-dependent kinase signaling.

HMGB1 also activates the non-canonical inflammasome pathway, leading to cell death through pyroptosis (10). This process can contribute to immune hyperactivity and immunosuppression, which may explain the late sepsis-related mortality. In hepatic infectious diseases, HMGB1 activity can be suppressed by glycyrrhizinic acid, providing a potential therapeutic strategy for modulating inflammation.

In severe pulmonary inflammatory diseases, including coronavirus disease-2019 (COVID-19), HMGB1 is abundantly secreted by necrotic pulmonary epithelial cells and innate immune cells (11). The disulfide form of HMGB1 triggers the release of pro-inflammatory cytokines, further exacerbating inflammation under these conditions.

## 4 Role of HMGB1 in cancer stem cell regulation

Cancer Stem Cell (CSCs) are a subpopulation of cells within a tumor that possess self-renewal and differentiation capabilities, similar to normal stem cells. These cells are thought to be responsible for tumor initiation, metastasis, and recurrence. In the context of CSC regulation, HMGB1 exhibits several functions that contribute to the maintenance and expansion of CSC populations.

HMGB1 promotes CSC self-renewal. By interacting with specific receptors and signaling pathways, such as LC3II/TLR4 associated transcriptional regulator (YAP1), HMGB1 stimulates the expression of genes involved in stem cell maintenance and proliferation (12, 13). This leads to an increase in the number of CSCs within the tumor, thereby enhancing the tumorigenic potential of cancer cells.

Following radiotherapy, differentiated cancer cells that regain stem cell characteristics can improve the population of CSCs, thus accelerating tumor recurrence and metastasis (14). This process involves the translocation of cytoplasmic YAP mediated by HMGB1/TLR2 into the nucleus. Subsequently, YAP forms a complex with hypoxia inducible factor 1 subunit alpha (HIF1A) in the nucleus, further activating dedifferentiation of CD133-negative pancreatic cancer cells.

## 5 Role of HMGB1 in metastasis and angiogenesis

HMGB1 overexpression and or elevated serum HMGB1 levels have been observed in several types of cancer, including nasopharyngeal carcinoma, liver cancer, prostate cancer, pancreatic cancer, melanoma, gastric cancer, esophageal cancer, cervical cancer, breast cancer, malignant pleural mesothelioma, and bladder cancer (15). This overexpression of HMGB1 is often associated with a poor prognosis. Jiao et al. indicated that HMGB1 plays a critical role in the invasion and metastasis of breast cancer (16). In particular, overexpression of HMGB1 can restore the migration ability of breast cancer cells, which is inhibited by silencing of the hematological and neurological expressed 1-like (HN1L) gene.

The tumor microenvironment (TME) is closely associated with tumor invasion and propagation (17). Neutrophils are crucial inflammatory cells in the environment (18). Tumor-infiltrating neutrophils promote glioma progression by regulating the HMGB1/RAGE/interleukin 8 (IL8) axis (19). HMGB1 is a crucial component of neutrophil extracellular traps and has strong affinity for RAGE receptors. It activates the nuclear factor kappa B (NF-κB) pathway, which effectively promotes the secretion of IL8, leading to increased proliferation of glioma cells. Continuous secretion of IL8 contributes to tumor angiogenesis and metastasis, thus enhancing the invasion of the TME by neutrophils.

The epithelial-to-mesenchymal transition (EMT) is a process driven by HMGB1, which may be an important factor responsible for tumor metastasis (20). HMGB1 up-regulates the expression of matrix metallopeptidases (MMPs), which further modulates the TME and results in EMT amplification. Additionally, a positive correlation has been revealed between metastasis to other sites and the activated RAGE/NF-κB signaling pathway, along with upregulated HMGB1 expression in prostate cancer cells.

Angiogenesis, the process of developing new blood vessels from preexisting capillaries, plays a vital role in various physiological and pathological processes, including tumor growth. In 2006, HMGB1 was identified as a factor that promotes angiogenesis in endothelial cells from various sources (21). Subsequent mechanistic studies revealed that RAGE receptors play a crucial role in extracellular HMGB1-mediated angiogenic activity through the mitogen-activated protein kinase/extracellular signal-regulated kinase (MAPK/ERK) pathway. Moreover, HMGB1 activates NF-κB through membrane receptor stimulation, leading to the production of angiogenic factors in hematopoietic cells (22).

Studies have demonstrated the upregulation of HMGB1 in cancerous tissues compared to that in surrounding non-malignant tissues. In breast cancer cells (MCF-7), HMGB1 promoted tumor vessel formation and enhances cancer cell migration by regulating HIF1A through the phosphatidylinositol 3 kinase/protein kinase B (PI3K/AKT) signaling pathway (23). The ability of HMGB1 to promote angiogenesis was also observed in ovarian cancer (24). Deacetylation and inhibition the cytosolic release of HMGB1 by sirtuin 1 (SIRT1) inhibits migration and angiogenesis in ovarian cancer cells (25).

Zhang et al. revealed a key role for HMGB1 in hepatitis B virus-induced primary liver cancer (26). During tumor formation, hepatitis B virus-encoded X protein (HBx) induced HMGB1 expression, which activates signal transducer and activator of transcription 3 (STAT3) to promote EMT and angiogenesis. Furthermore, there was a reciprocal relationship between HMGB1 and miR-34a expression levels. HBx-induced HMGB1 expression was regulated by the NF-κB signaling pathway, which was blocked by miR-34a, whereas STAT3 decreased miR-34a expression. Furthermore, extracellular HMGB1 activated the IL6/STAT3/miR-34a axis.

In gastric cancer, IL8 is the most notable cytokine associated with serum HMGB1; suppression of IL8 markedly reduces human umbilical vein endothelial cell migration and tubule formation activities induced by recombinant human HMGB1 or mock-transfected HMGB1 (27). These findings suggest that HMGB1 overexpression promotes tumor angiogenesis through IL8 expression in gastric cancer.

In conclusion, HMGB1 plays a crucial role in tumor angiogenesis by activating various signaling pathways and inducing the production of angiogenic factors. The identification of HMGB1 as a promoter of angiogenesis in cancerous tissues provides a promising therapeutic target for cancer treatment.

## 6 Role of HMGB1 in autophagy

**Figure 2:**
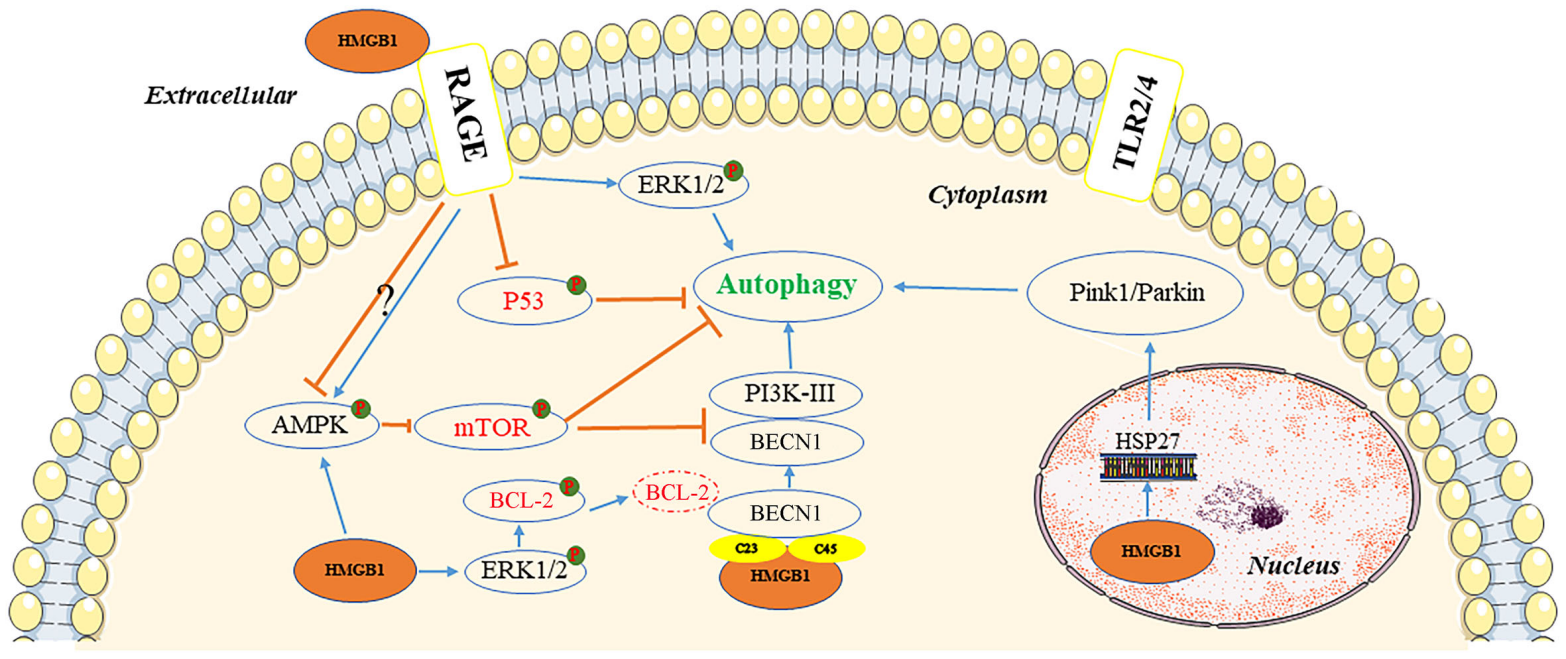
Pathways of autophagy induction by HMGB1. In the extracellular environment, reduced-HMGB1 could bind to the RAGE receptor rather than TLR2/4 to activate ERK1/2 and inhibit the expression of TP53, or exert an effect on the AMPK/mTOR pathway. In the cytoplasm, HMGB1 could induce the dissociation of the BECN1/BCL2 complex by ERK1/2 or bind to BECN1. It also activated the AMPK/mTOR pathway. In the nucleus, HMGB1 could stimulate HSP27 transcription to lead to autophagy via the PINK1/Parkin pathway. AMPK, AMP-activated protein kinase; BCL2, B-cell CLL/lymphoma 2; BECN1, beclin 1; ERK, extracellular signal-regulated kinase; HMGB1, high mobility group box 1; HSP27, heat-shock protein 27; mTOR, mechanistic target of rapamycin kinase; PI3K, phosphatidylinositol 3 kinase; PINK1, PTEN induced kinase 1; RAGE, receptor for advanced glycation end products; TLR, toll-like receptor; TP53, tumor protein 53.

Autophagy is a cellular self-degradation mechanism closely associated with cancer metabolism and metastasis (28). HMGB1 may be a key protein in the induction of this process (29). The protein can induce autophagy through several pathways, depending on its location within the cell (**Figure 2**).

Inside the nucleus, HMGB1 modulates the expression of the heat-shock protein 27 (HSP27) gene and activates PTEN induced kinase 1/Parkin (PINK1/Parkin), an important pathway that regulates mitochondrial autophagy and acts as a transcription factor. Furthermore, HSP27 phosphorylation regulates actin polymerization and contributes to mitophagy (30). It is not clear whether HSP27 expression is regulated by HMGB1, or whether HSP27 is required for HMGB1-dependent mitophagy. To initiate mitophagy, PINK1 generates phospho-ubiquitin Parkin and promotes its mitochondrial translocation, which participates in mitochondrial autophagy by mediating voltage dependent anion channel 1 (VDAC1) ubiquitination. In this process, p62 is transported to the mitochondria, where it binds to the microtubule associated protein 1 light chain 3 alpha (MAP1LC3), leading to mitochondrial autophagy (31). PINK1-induced autophagy may also be Parkin-independent, and optineurin (OPTN) and NDP52 can stimulation of unc-51 like autophagy activating kinase 1 (ULK1), DFCP1, WD repeat domain, and phosphoinositide interacting 1 (WIPI1) protein molecules, inducing the mitochondrial degradation. Parkin functions as an amplifier that enhances PINK1-triggered mitochondrial autophagy signaling (31).

HMGB1 has also been documented as a beclin 1-binding (BECN1-binding) protein, which plays an important role in sustaining autophagy in the cytoplasm. It can bind to BECN1 through an intramolecular disulfide bridge (C23/45), resulting in dissociation of the inhibitory partner of BECN1 B-cell CLL/lymphoma 2 (BCL2). Tang et al. demonstrated that mutations in C23S and C45S led to the loss of the ability of HMGB1 to bind to BECN1, consequently inhibiting autophagy (32). Furthermore, phosphorylation of BCL2 by HMGB1 through the activation of the ERK/MAPK pathway may also be a possible mechanism involved in the inhibition of the BECN1/BCL2 complex (33). Subsequently, the BECN1/phosphatidylinositol 3-kinase catalytic subunit type 3 (BECN1/PIK3C3) complex is formed, initiating the recruitment of autophagy related (ATG) proteins to the phagophore to induce autophagy.

Extracellular HMGB1 due to tumor cell death can induce autophagy, with RAGE receptors playing a central role in HMGB1-induced autophagy. The redox state of HMGB1 is a crucial factor in determining its pathophysiological activities, with reduced binding of HMGB1 to RAGE receptors promoting autophagy; although, oxidized HMGB1 did not exert an effect (33). HMGB1 can regulate autophagy through a HMGB1-RAGE-ERK1/2-dependent pathway, enabling survival of Lewis cells (34). The mechanistic target of rapamycin kinase (mTOR), a serine/threonine kinase, also plays an important role in the regulation of autophagy (35). HMGB1, stimulated by chemotherapeutic drugs, can be transported to the cytoplasm and promotes autophagy through activation of AMP-activated protein kinase (AMPK), which in turn causes inhibition of mTOR in hepatocellular carcinoma cells (HCC) (36). The AMPK/mTOR pathway can act downstream of HMGB1/RAGE to regulate autophagy in HCC (36). However, RAGE has a negative regulatory effect on the AMPK/mTOR signaling pathway.

Tumor protein 53 (TP53) is a tumor suppressor with a wide range of functions. The function of TP53 in autophagy regulation is strongly influenced by its subcellular location. In a transcription-independent manner, cytoplasmic TP53 protein suppresses autophagy by inhibiting HMGB1/BECN1 complex formation (36). TP53 knockout enhanced autophagy and promoted the translocation of HMGB1 from the nucleus to the cytoplasm. Lai et al. reported that extracellular binding of HMGB1 to RAGE receptor exerted an inhibitory effect on TP53, which was conducive to autophagy (37).

## 7 Role of HMGB1 in chemotherapy and radiotherapy resistance

Apoptosis induced by chemotherapy drugs or radiotherapy is an important type of cell death kills cancer cells. However, changes in TME and antiapoptotic factors often result in the development of drug resistance, leading to treatment failure and cancer relapse. HMGB1 generally plays a blocking role during the treatment process. In this section, we discuss the role of HMGB1 in the regulation of certain commonly used anticancer drugs.

### 7.1 Adriamycin

Li et al. revealed that adriamycin significantly increases HMGB1 levels in the cytoplasm of HCC cells. HMGB1 enhances autophagy and protects cancer cells from adriamycin-induced apoptosis by activating the AMPK/mTOR signaling pathway, which further contributes to chemoresistance in HCC cells (36). Lai et al. found that RAGE activation though HMGB1 binding was involved in drug resistance, including resistance to adriamycin in acute leukemia. This occurred through three mechanisms: 1) HMGB1 enhanced autophagy by activating ERK signaling, thus inhibiting mTOR activation and initiating the activity of the ULK1/ATG13/FIP200 complex; 2) HMGB1 inhibited apoptosis by restricting the BCL2/BCL2 associated X (BCL2/BAX) pathway with TP53 involvement; and 3) HMGB1 activated the NF-κB pathway, thereby inducing the expression of P-glycoprotein (P-gp) and multidrug resistance-associated protein (MRP) to expedite drug excretion (37). Similarly, Huang et al. confirmed the role of HMGB1 in multidrug resistance, including resistance to adriamycin, by regulating the formation of the BECN1/class III phosphatidylinositol 3-kinase (BECN1/PI3KC3) complex involved in autophagy in osteosarcoma (38, 39). Furthermore, resistance to adriamycin caused by HMGB1 has also been found in breast cancer (40, 41) and neuroblastoma (42).

### 7.2 Cisplatin

Zhu et al. confirmed that HMGB1 expression is markedly increased in resistant nasopharyngeal carcinoma cells compared to that in sensitive cells (43). Mechanistic studies substantiated HMGB1-induced IL6 expression followed by activation of the Janus kinase-STAT3 (JAK-STAT3) pathway, which results in acquired resistance to cisplatin. In addition, studies have confirmed that long noncoding RNA myocardial infarction associated transcript (MIAT) is an important regulatory factor of HMGB1. In another study, Zhu et al. demonstrated that HMGB1 interaction with Ku autoantigen, and KU70 participates in resistance to cisplatin and ionizing radiation (IR) by promoting the efficiency of non-homologous end joining (NHEJ) (44). HMGB1 may be associated with the development of resistance to cisplatin in lung adenocarcinoma cells. Higher levels of HMGB1 have also been detected in cisplatin-resistant A549/DDP cells compared to cisplatin-sensitive A549 cells (45). In cisplatin-persistent 1.3.11, A549, HCC4006, and SKLU-1 cells, the expression of RAGE was increased, while that of TLR-2 or TLR-4 was not significantly changed (46). Furthermore, downregulation of HMGB1 by short hairpin RNA (shRNA) helped restore the chemosensitivity of lung cancer cells.

### 7.3 Vinblastine

Zhan et al. showed that extracellular HMGB1 displayed cytoprotective activity against SGC-7901 and BGC-823 gastric cancer cells using the microtubule-targeting drug vincristine. The drug induces the mitochondrial apoptotic pathway via transcriptional upregulation of myeloid cell leukemia 1 (MCL1), an anti-apoptotic member of the BCL2 protein family (47). Inhibition of RAGE expression by small-interfering RNA (siRNA) resulted in the blockage of MCL1 mRNA upregulation induced by recombinant HMGB1 activation. This led to enhanced induction of apoptosis by vincristine. Additionally, the knockdown of TLR2 or TLR4 partially affected the regulation of recombinant HMGB1. In K562 cells, HMGB1 can induce autophagy and increase vinblastine chemotherapeutic resistance via the AMPK-mTOR pathway (48). Extracellular HMGB1 has been shown to considerably increase P-gp expression in human gastric adenocarcinoma cells, thus, increasing resistance to adriamycin and vincristine and encouraging the development of multidrug resistance (49).

### 7.4 Taxane

Lei et al. observed constant paclitaxel-induced expression of HMGB1 in metastatic castration-resistant prostate cancer cells. Furthermore, HMGB1 can activate c-Myc signaling, an important pathway that contributes to drug resistance in various types of cancers (50). Similarly, several reports showed that protective autophagy induced by HMGB1 is also an important mechanism for resistance to taxane (51, 52).

### 7.5 Radiation therapy

Radiation therapy is an essential treatment option for cancer. The generation of extracellular neutrophil traps induced by HMGB1, an important inhibitor of radiotherapy, has been observed in bladder cancer models mediated by TLR4 expression and has been confirmed by tumor growth regression through inhibition of HMGB1 (53). In contrast, HMGB1 may promote the sensitivity of tumor cells to radiotherapy. HMGB1 suppression increases cell proliferation and invasion, but decreases sensitivity to radiation therapy in cervical cancer, and HMGB1 induction exerts the opposite effects (54). The enhancing effect of HMGB1 on sensitivity to radiotherapy may be related to increased expression of retinoblastoma (RB), a tumor suppressor protein.

Exposure to ultraviolet (UV) radiation is an important risk factor for melanoma development. The discovery of an autonomous cell response to UV damage by the HMGB1/RAGE pathway in melanocytes may contribute to their resistance to apoptosis and cell death, and may have implications for the early stages of melanoma formation (55).

### 7.6 Other pathways involving HMGB1

Yin et al. demonstrated that HMGB1 counteracts the cytotoxicity of gemcitabine by mediating the c-Jun N-terminal kinase (JNK) and ERK activities to induce protective autophagy. Knockdown of HMGB1 strongly enhances gemcitabine-induced apoptosis in bladder cancer cells and suppresses autophagy (56). Furthermore, gemcitabine resistance may be related to activation of HSP27 (an important drug resistance-related protein) by HMGB1 (57, 58).

Higher levels of HMGB1 expression have also been found in chemotherapy-resistant RPMI8226/ADR, RPMI8226/BOR, and RPMI8226/DEXMM cell lines. Silencing endogenous HMGB1 using shRNA improved drug sensitivity and decreases NF-κB signaling activity in chemotherapy-resistant MM cells. Activation of NF-κB signaling reverses the enhancement of drug sensitivity caused by HMGB1 silencing, indicating that the NF-κB signaling pathway is critical in drug resistance mediated by HMGB1 (59).

**Figure 3:**
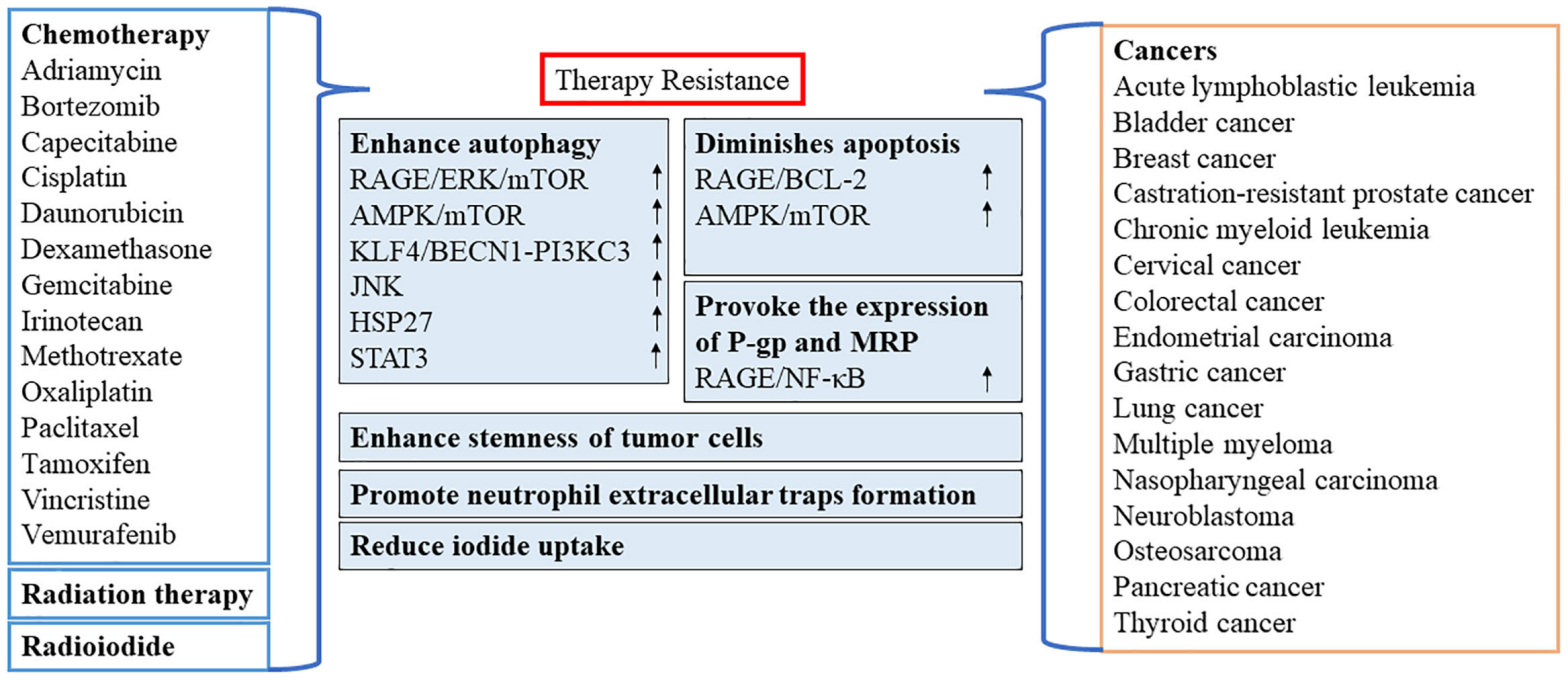
Role of HMGB1 in resistance to chemotherapy or radiation therapy. AMPK, AMP-activated protein kinase; BECN1, beclin 1; BCL2, B-cell CLL/lymphoma 2; ERK, extracellular signal-regulated kinase; HSP27, heat-shock protein 27; STAT3, signal transducer and activator of transcription 3; JNK, c-Jun N-terminal kinase; KLF4, KLF transcription factor 4; MRP, multidrug resistance-associated protein; mTOR, mechanistic target of rapamycin kinase; NF-κB, nuclear factor-kappa B; P-gp, P-glycoprotein; PI3KC3, class III phosphatidylinositol 3-kinase; RAGE, receptor for advanced glycation end products.

In recent years, numerous reports have confirmed the importance of HMGB1 as a target for chemotherapy or as a resistance to radiation therapy in various types of cancer (**Figure 3**). However, oxidized HMGB1 enhances the cytotoxicity of chemotherapeutic agents and cause apoptosis through the caspase 9/3 (CASP9/3) intrinsic pathway (60). Therefore, it is of great significance to identify the redox state and subcellular location of HMGB1. This would allow researchers to better understand whether HMGB1 inhibits or promotes apoptosis by inducing protective autophagy, blocking or activating CASP-dependent pathways may be optimal as a clinical antitumor strategy.

## 8 Role of HMGB1 in necrosis

A growing body of evidence indicates that necrosis is a molecular control mechanism and a modulated form of cell death rather than simply an uncontrolled and accidental process. The concept of regulatory necrosis currently encompasses several modes of cell death, such as oncosis, necroptosis, ferroptosis, and pyroptosis. Unlike tumor-suppressed apoptosis or autophagic cell death, necrosis is considered ‘repair cell death’ occurring during tumor progression and invasion. The cell membrane of necrotic cells is ruptured, and cytoplasmic contents such as HMGB1 are released into the extracellular space (61, 62). Apoptotic cells modify their chromatin to bind to HMGB1 due to generalized histone acetylation, to prevent the release of HMGB1 in the early stages. In contrast, necrotic cells release HMGB1 passively through a special vesicle-mediated secretory pathway; independently of the endoplasmic reticulum-Golgi apparatus because of the lack of a signal peptide (63).

HMGB1 is involved in tumor progression as a pro-inflammatory and protumor cytokine released by necrotic cells (62). Liu et al. confirmed that etoposide caused the release of HMGB1 in acute myeloid leukemia cells. However, this extracellular HMGB1 prevented etoposide-induced necroptosis through the NF-κB pathway rather than preventing the degradation of the cellular inhibitor of apoptosis 1/2 (cIAP1/2) or X-linked inhibitor of apoptosis (XIAP) (64).

Iron-dependent lipid peroxidation is a characteristic of ferroptosis, a newly identified type of controlled necrosis. Wen et al. demonstrated that HMGB1 is a DAMP released by ferroptotic cells and positively regulated by an autophagic pathway dependent on ATG5- and ATG7 (65). Histone deacetylase (HDAC) inhibitors, which can induce autophagy and enhance HMGB1 acetylation, can lead to the release of HMGB1 in ferroptosis. Current study has showed that RAGE rather than TLR4 is required for the production of TNF mediated by HMGB1 in macrophages in response to ferroptotic cells. This evidence implies that inhibition of the HMGB1/RAGE pathway could reduce ferroptosis-induced inflammation. Erastin, a ferroptosis inducer, promotes cytoplasmic translocation of HMGB1 in HL-60/NRASQ61L cells. Ye et al. found that HMGB1 knockdown could effectively suppressed iron-mediated ROS generation and erastin antitumor activity through the RAS-JNK/p38-dependent signaling pathway (66). These findings imply that HMGB1-mediated ferroptosis may be a potential target for cancer therapy.

Pyroptosis is a type of programmed cell necrosis that is mainly mediated by pro-inflammatory gasdermin (GSDM). The N-terminus of the cleaved GSDM generates pores in the cell membrane during pyroptosis, resulting in cell disintegration and the release of intracellular factors, such as HMGB1 (67). Tan et al. observed that HMGB1 and GSDME-mediated pyroptosis released could promote the development of colorectal cancer (68). This study further revealed that HMGB1-induced activation of ERK1/2 signaling leads to colorectal cancer carcinogenesis and proliferating cell nuclear antigen (PCNA) expression, which is a key proliferation marker indicating the rate at which cells multiply.

## 9 Role of HMGB1 in immunotherapy

### 9.1 Immunogenic cell death

Immunogenic cell death is a mode of tumor cell death caused by the activation of the host immune system in response to chemotherapy, UV radiation, photodynamic therapy, or radiotherapy (69). This activation involves the release of DAMPs, such as calreticulin (CALR), HMGB1, adenosine triphosphate, and HSPs, from dead tumor cells (70, 71). Cytotoxic T cells are recruited by antigen-presenting cells to kill cancer cells directly. HMGB1 plays an important role in immunogenic cell death, while acting as a well-characterized DAMPs. However, as already discussed, the redox state of HMGB1 plays a critical role in its immunogenic activity of HMGB1 (72). Redox modifications regulate the translocation, release, and activity of HMGB1. The fully reduced HMGB1 translocated to extracellular space following injury has chemokine activity, recruiting immune cells. Extracellular immune cells can directly secrete disulfide-HMGB1, or they can oxidize reduced all-thiol-HMGB1 to disulfide-HMGB1 by ROS generation. Disulfide-HMGB1 alone has cytokine activity; it promotes the release of pro-inflammatory cytokines, thus participating in the inflammatory response. Finally, HMGB1 is oxidized by ROS to become all-oxidized-HMGB1, consequently abolishing its cytokine and chemokine activities. Although HMGB1 can be released both actively and passively, no significant difference was observed in the biological and molecular events mediated by this release (73).

A main mechanism through which HMGB1 acts its role as a DAMP is the activation of TLR4 and its adaptor myeloid differentiation primary response protein (MYD88) in dendritic cells (DCs). This activation underlies the processing and presentation of dying tumor cell antigens. Silencing of HMGB1 with siRNA in the doxorubicin-treated CT26 or MCA205 tumor model and irradiated EG7 dying tumor cells disrupts antigen presentation and inhibits the priming of T cells (74). Furthermore, HMGB1 promotes DC activation and elicits an immune response through activation of the RAGE receptor. Owing to the chemokine receptors C-C motif chemokine receptor 7 (CCR7) and C-X-C motif chemokine receptor 4 (CXCR4), lipopolysaccharide-stimulated DCs can migrate to CCL19 and CXCL12. However, the presence of antiHMGB1 and antiRAGE antibodies inhibits the migration of lipopolysaccharide-stimulated DCs (75). The role of extracellular HMGB1 in promoting DC activation and recruitment of T cells has also been demonstrated in clinical studies in addition to preclinical models (76). A study analyzing tissue samples obtained from patients with non-small cell lung cancer showed that high expression of HMGB1 and CXCL11 was positively correlated with overall survival of patients. Similarly, plasma HMGB1 levels were significantly higher in patients with breast cancer who were sensitive to epirubicin/docetaxel than in non-responders (77). The above data suggest that radiation therapy and chemotherapy-induced elevation of HMGB1 expression may be a favorable prognostic tool, and HMGB1 may be an indispensable regulatory factor in immunotherapy.

### 9.2 M1 polarization

Macrophages are widely distributed white blood cells that are activated under different inflammatory conditions, and include M1 (pro-inflammatory) and M2 (anti-inflammatory) macrophages, under appropriate stimulation. By upregulating the expression of pro-inflammatory mediators and creating an inflammatory state, M1 macrophages play an important role in tumor suppression. In contrast, M2 macrophages contribute to disease development by secreting anti-inflammatory cytokines (78). Macrophages interact with tumor cells and other components in the TME and undergo polarization to the M2-type, thus exhibiting different tumor-promoting actions. M1 polarization was induced by recombinant HMGB1 or the MAPK-p38 stimulator, as reported by He et al. (79). Positive-feedback release or production of HMGB1 and RAGE via the MAPK-ERK pathway in macrophages promoted M1 macrophage polarization. Li et al. also reported that M1-like polarization of tumor-related macrophages was aided by the HMGB1/RAGE/NF-κB/NLR family pyrin domain containing 3 (HMGB1/RAGE/NF-κB/NLRP3) pathway, and elevated levels of HMGB1 enhanced the sensitivity of glioblastoma cells to temozolomide (80).

### 9.3 Chemotherapy-induced peripheral neuropathy

Although the inflammatory response induced by HMGB1 is helpful in tumor immunotherapy, its inflammatory side effects should not be underestimated. The pro-inflammatory effect of HMGB1 is reflected in tumor immunotherapy and is a potential therapeutic target for several inflammatory diseases, including COVID-19 (81). Chemotherapy-induced peripheral neuropathy (CIPN) is a common complication of chemotherapeutic drugs such as paclitaxel, cisplatin, and vincristine, which can have adverse effects on the therapeutic efficiency and quality of life of patients. In severe cases, dosage reduction or discontinuation of chemotherapy may be necessary. In recent years, numerous studies have investigated the pharmacological effects of HMGB1 on tumor pain and neuralgia, given its strong pro-inflammatory and propain activities (82–84). Studies have shown that an anti-HMGB1-neutralizing antibody can effectively inhibit CIPN in animals receiving chemotherapy (85). Similarly, recombinant human soluble thrombomodulin, an antibody that inactivates HMGB1, also prevents the development of CIPN in rats treated with paclitaxel or vincristine (86). Overall, HMGB1 mainly mediates CIPN induced by inflammatory factors through the activation of receptors, such as RAGE, TLRs, and CXCR4 on the cell membrane, since macrophages actively secreted HMGB1 after paclitaxel stimulation (85).

### 9.4 Immunotolerance

Numerous studies have demonstrated the vital role of HMGB1 in immune cells activations. However, recent investigations have shown that several components neutralize extracellular HMGB1 or alter its pro-inflammatory to anti-inflammatory effects (87). HMGB1 contributes to protumor action of the M2 macrophage phenotype in a RAGE-dependent manner. Hypoxia-induced HMGB1 production promotes the accumulation of M2-like macrophages and an IL10-rich environment through RAGE signaling (88). C1q/leukocyte-associated immunoglobulin like receptor 1 (Clq/LAIR1) is one of the most important associates of HMGB1/RAGE for inducing anti-inflammatory activities. C1q is an evolutionarily conserved molecule with immunosuppressive effects, and LAIR1 (an immunoglobulin superfamily transmembrane protein) is a high-affinity receptor for C1q. These four proteins form a polymeric protein complex, which causes monocytes to adopt an M2-like phenotype. This process increases the production of CD163 and several anti-inflammatory molecules, including programmed cell death 1 ligand 1 (PDL1), Mer, and IL10 (89). However, the immunogenic activity of HMGB1 is dependent on its redox state. A clinical study focusing on basal-like breast cancer cells reported the release of high levels of HMGB1. Moreover, the tumor-specific cytoplasmic expression of HMGB1 is further linked to immunological tolerance and poor clinical outcomes. In cytoplasmic HMGB1-positive malignancies, an increased density of CD206^+^ M2 macrophages has been found in the TME, along with amplification in forkhead box P3 (FOXP3^+^) regulatory T cells (90). Furthermore, inhibition of extracellular HMGB1 decreases the growth of preexisting solid tumors and enhances the therapeutic effectiveness of anti-programmed cell death 1 (anti-PD1) treatment in immunocompetent mice by activating anticancer immune responses. In a highly oxidized TME, the reduced-disulfide/oxidized ratio of HMGB1 may be less than 2:1. Furthermore, DC activation and its tolerogenic activity were considerably increased by the reduced and oxidized forms of HMGB1, which can be negatively regulated by RAGE inhibition.

Ye et al. on HCC-derived exosomes confirmed that HMGB1 can trigger the production of protumorigenic regulatory B cells (Breg) (89). T-cell immunoglobulin mucin family member 1 (TIM1^+^) Breg cells appear to include the highest number of IL10-producing B cells; these cells have 8–20 times higher IL10 expression than the rest of the B cell subsets. In addition, they account for >70% of all IL10-producing B cells (90). The IL10 produced by these TIM1^+^ Breg cells was sufficient to induce tremendous anti-immunogenic activity. Initially, through the HMGB1/TLR2/4/MAPK pathway, HCC cells release exosomes that can stimulate the accumulation of TIM1^+^ Breg cells. Subsequently, by secreting IL10 and restricting CD8^+^ T cell activities, TIM1^+^ Breg cells generate an immunosuppressive milieu that promotes HCC growth.

## 10 Therapeutic targeting of HMGB1

Currently, there are no commercially available drugs that specifically target HMGB1 expression. Nevertheless, several candidates are currently in clinical development. CD24Fc, a first-in-class recombinant fusion protein, serves as an immunomodulator targeting a novel immune checkpoint in the innate immune system (91). It inhibits HMGB1, HSP70/90, and other molecules. In particular, CD24Fc has demonstrated promising activity for symptomatic improvements in patients with severe COVID-19 (92). Although Merck Sharp & Dohme (NJ, USA) has discontinued its development as a treatment for COVID-19, CD24Fc retains its potential usefulness in the treatment of malignancies such as melanoma.

SB17170 and SB1703 are small-molecule drugs developed by Spark BioPharma (Seoul, RK) that specifically target HMGB1. In particular, SB17170 served as a prodrug, for its active metabolite SB1703. SB17170 exhibited antitumor activity in B16F10 murine syngeneic models, but not in immunodeficient models, indicating that its antitumor mechanism relies on the participation of the immune system. Treatment with SB17170 induced intratumoral T cell infiltration, reduced the number of MDSC, and lowered the levels of HMGB1 in the blood. When used in combination with immune checkpoint inhibitors, SB17170 enhances antitumor responses (93).

Dociparstat sodium (DSTAT, CX-01) is a low anticoagulant heparin with multiple mechanisms of action, including inhibition of the CXCR4/CXCL12 axis, blockage of HMGB1, and binding of platelet factor 4 (PF-4) (94). Its therapeutic effect in combination with standard chemotherapy for acute myeloid leukemia is currently under investigation in phase III clinical trials.

## 11 Conclusion

HMGB1 is a multifunctional molecule that in intricately involved in various pathological conditions, particularly in inflammatory disorders and malignancies. It plays a pivotal role in numerous biological processes and significantly promotes the development, migration, and angiogenesis of tumors in both the extracellular and endogenous environments. This promotion is achieved through the binding of its ligand to receptors such as RAGE and TLR4. Furthermore, HMGB1 has been implicated in the emergence of resistance to antitumor drugs, either by inducing protective autophagy or by enhancing the expression of drug-resistant proteins.

As a DAMP, HMGB1 effectively in recruits DCs. In addition, it can induce immunogenic cell death, thereby activating the immune response of the body. However, it should be noted that the cytokine and chemokine activities of reduced-HMGB1 are often triggered by oxidative agents (e.g., ROS) in the TME.

Considering the complex and multifaceted nature of HMGB1, future studies must investigate several crucial aspects. First, it is important to clarify the specific role of each HMGB1 receptor in the immune response. This understanding will help us gain insight into the mechanisms by which HMGB1 interacts with and modulates the immune system. Second, understanding the dynamics of HMGB1 translocation between the intracellular and extracellular environments is essential. This knowledge could provide valuable clues on the functions of HMGB1 in different cellular compartments and its potential contribution to pathological processes.

It remains unclear whether the release of HMGB1 during different types of cell death mediates similar immune responses. To our knowledge, this is a crucial gap that must be addressed. In addition, the interaction between HMGB1 and non-immunogenic DAMPs in inflammatory regulation requires further investigation.

Finally, the development of effective and selective HMGB1 inhibitors remains a critical research topic. Such inhibitors, if designed to specifically target the intracellular or extracellular functions of HMGB1, could potentially enhance tumor therapy by blocking autophagy and nuclear homeostasis. This approach holds promise for the development of novel antitumor medications.

In conclusion, HMGB1 is a complex and multifaceted molecule with widespread implications in various pathological conditions. Future research should focus on clarifying its specific roles and interactions in the immune response, translocation dynamics, and inflammatory regulation to improve our understanding of its role in tumor growth and progression. The development of targeted inhibitors of HMGB1 represents a promising avenue for antitumor therapy.

## Author contributions

GL: Writing – original draft, Writing – review & editing. MY: Writing – original draft, Writing – review & editing. KG: Visualization, Writing – original draft. QJ: Writing – review & editing. ZW: Writing – review & editing. BW: Conceptualization, Supervision, Writing – review & editing. XL: Conceptualization, Supervision, Writing – review & editing.
